# Successful administration of sequential TVEC and pembrolizumab followed by Temozolomide in immunotherapy refractory intracranial metastatic melanoma with acquired B2M mutation

**DOI:** 10.18632/oncotarget.27848

**Published:** 2020-12-29

**Authors:** Karam Khaddour, Joshua Dowling, Jiayi Huang, Martha Council, David Chen, Lynn Cornelius, Tanner Johanns, Sonika Dahiya, George Ansstas

**Affiliations:** ^1^Department of Medicine, Division of Medical Oncology, Washington University School of Medicine, St. Louis, Missouri, USA; ^2^Department of Neurological Surgery, Washington University School of Medicine, St. Louis, Missouri, USA; ^3^Department of Radiation Oncology, Washington University School of Medicine, St. Louis, Missouri, USA; ^4^Division of Dermatology, Washington University School of Medicine, St. Louis, Missouri, USA; ^5^Division of Neuropathology, Department of Pathology and Immunology, Washington University School of Medicine, St. Louis, Missouri, USA

**Keywords:** metastatic melanoma, immune checkpoint inhibitor, temozolomide, acquired resistance, beta-2 microglobulin

## Abstract

Despite the substantial advances in the management of metastatic melanoma with the introduction of immune checkpoint inhibitors (ICI), many patients develop disease progression during treatment with immunotherapy. This has been suggested to be mediated by several mechanisms that contribute to acquired resistance to ICI, one of which is acquired beta-2 microgloubulin (B2M) mutation. Talimogene laherparepvec (TVEC) is a genetically modified oncolytic virus that can enhance antitumor immunity. Temozolomide (TMZ) is an oral alkylating agent that has been suggested to augment anti-tumor immune response. The clinical significance of TVEC and TMZ in metastatic melanoma patients who are refractory to immunotherapy is unknown. We report a case of a patient with immunotherapy refractory intracranial metastatic melanoma after initial response to ICI who had acquired B2M mutation. The patient received TVEC and pembrolizumab followed by TMZ. The patient maintained durable response of her visceral and intracranial disease for 19 months and ongoing. More research is essential to delineate whether TVEC or TMZ has efficacy in immunotherapy refractory metastatic melanoma with acquired B2M mutation.

## INTRODUCTION

The development of immune checkpoint inhibitors (ICI) has transformed our management of metastatic malignancies and reshaped the field of oncology. ICIs are monoclonal antibodies that target checkpoint receptors present on tumor and immune cells in the tumor microenvironment. These medications unleash the anti-tumor immune response by targeting checkpoint receptor proteins such as programmed death-1 (PD-1), programmed death ligand-1 (PD-L1) or cytotoxic associated lymphocyte antigen-4 (CTLA-4), among other receptors [1]. For metastatic melanoma, ICIs were approved after clinical trials demonstrated improved progression free survival (PFS) and overall survival (OS) over standard of care, and are now considered first line therapy. The long-term follow-up studies in patients with metastatic melanoma who were treated with ICI have further supported the durable responses achieved. The 5-year survival data with combined nivolumab (PD-1 inhibitor) and ipilimumab (CTLA-4 inhibitor) in the CheckMate 067 study has shown a median OS of more than 60 months in patients with metastatic melanoma [2]. Similarly, the 5-year follow up in the KEYNOTE-001 trial of patients with metastatic melanoma who were treated with pembrolizumab (PD-1 inhibitor) showed a median OS of 38.6 months in treatment-naïve patients [3]. Despite the remarkable results observed in multiple clinical trials, a significant number of patients develop progressive disease during treatment [2, 3]. This has been suggested to be mediated by acquired resistance in the tumor microenvironment [4]. Of importance to this report, mutations in the beta-2-microglobulin (B2M), which is an essential component of MHC class I antigen presentation, have been suggested to confer acquired resistance to PD-1 inhibitors in metastatic melanoma [5]. Talimogene laherparepvec (TVEC) is a genetically modified oncolytic virus that has been suggested to restore tumor sensitivity to ICI and overcome resistance in immunotherapy refractory melanoma [6, 7]. Temozolomide (TMZ) which is an oral alkylating agent has demonstrated modest efficacy in the treatment of melanoma prior to the era of targeted therapy and ICI [8]. Interestingly, there is evidence of the modulating effect of TMZ on T-cell subsets which could alter the balance of the immune system but this has not been well studied in melanoma [9]. Here, we report a case of a patient who presented with recurrent metastatic melanoma that initially responded to ICI but later progressed with identification of an acquired B2M mutation (loss of function) among other mutations that were detected. The patient was treated with TVEC and pembrolizumab followed by TMZ. The patient had complete durable remission for 19 months and ongoing. We discuss the possible role of TVEC and TMZ in immunotherapy resistant metastatic melanoma based on the available literature regarding their modulating effect of the immune system.

## CASE PRESENTATION

**Figure 1:**
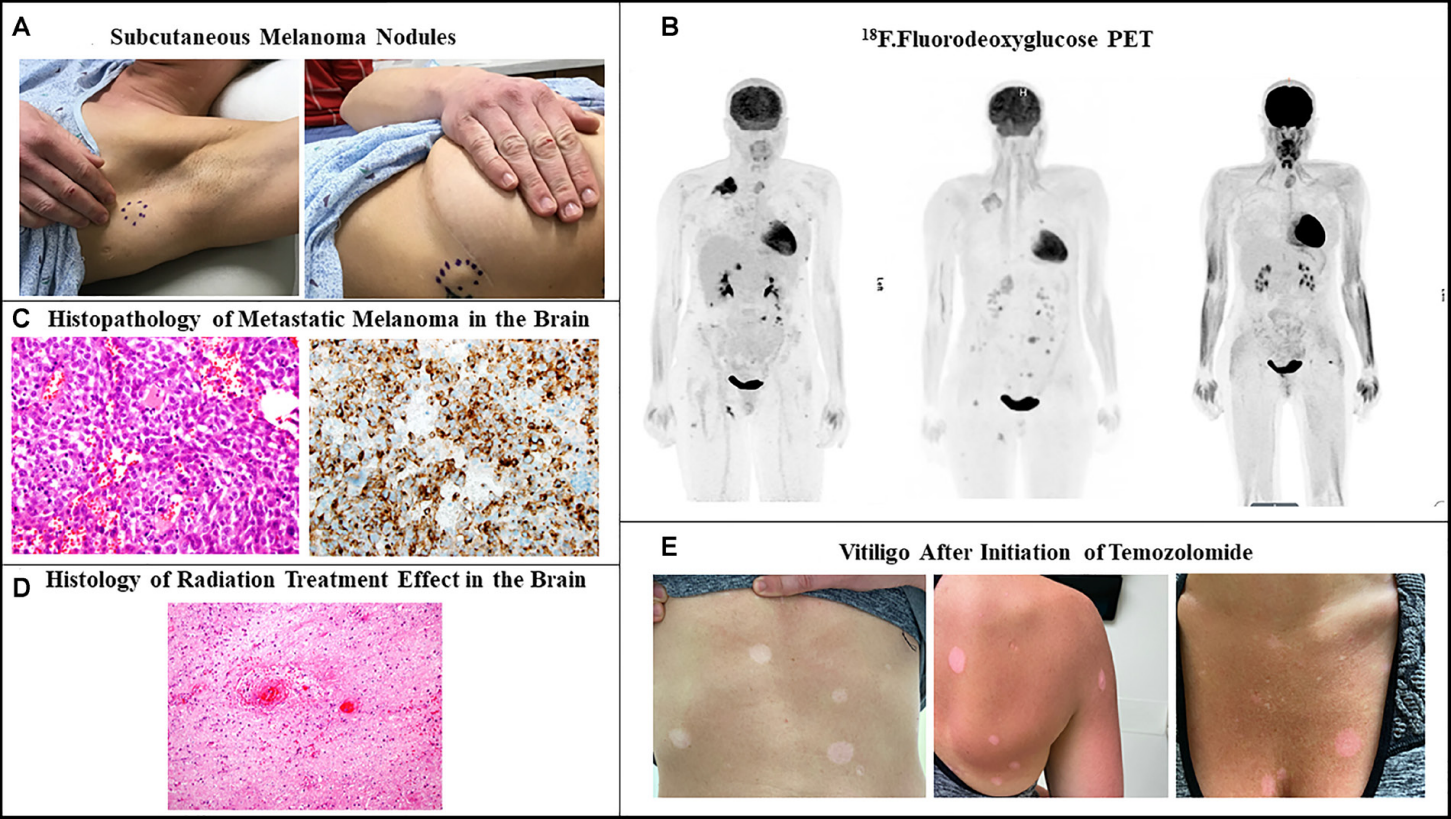
Recurrent metastatic melanoma. (**A**) Firm subcutaneous nodules in the axillary area and lateral chest wall. (**B**) ^18^F.Fluorodeoxyglucose positron emission tomographic scan showing multiple metastatic lesions in the subcutaneous tissue, lung, breast, adrenal gland, pelvic and perianal area before start of ipilimumab-nivolumab (left). Persistent hypermetabolic metastatic lesions with increase in size an appearance of new lesions after 3 months of ipilimumab-nivolumab (center). Complete metabolic response one year after temozolomide (right). (**C**) Histopathology of metastatic brain lesions showing in the left malignant cells with an epithelioid appearance, harboring vesicular nuclei, and prominent nucleoli (H&E, 40X). In the right picture immunostain for HMB45 demonstrates strong diffuse cytoplasmic to paranuclear Golgi staining pattern in the tumor cells (B; 40X). (**D**) histopathology of another resected brain lesion showing reactive changes, hyalinized vessels, bland necrosis, and no evidence of malignancy Post TVEC and Pembrolizumab (consistent with pseudoprogression). (**E**) Hypo-pigmented macules and patches on the back, anterior chest wall and abdomen.

**Figure 2:**
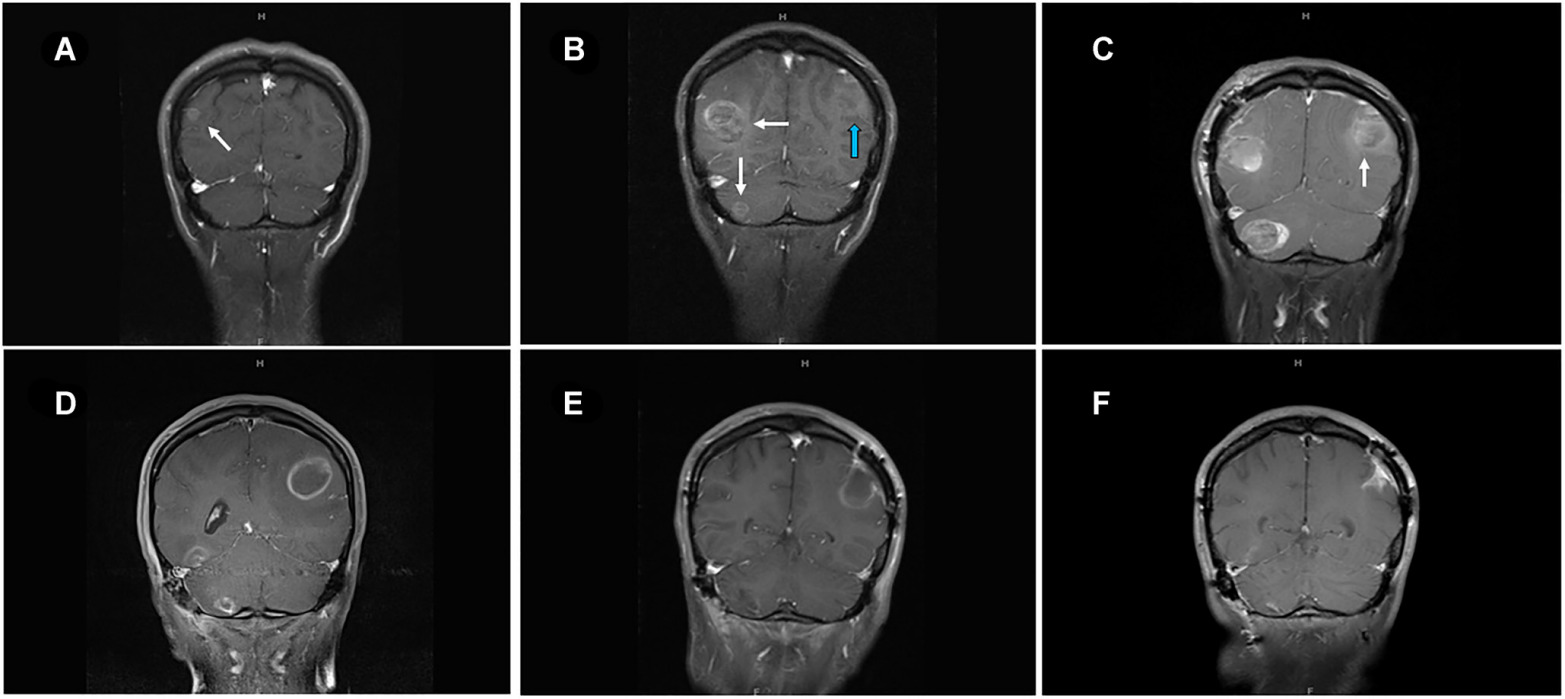
MRI of brain of metastatic lesions showing (**A**) coronal section demonstrating right parieto-occipital lesion at the start of ipilimumab-nivolumab (arrow). (**B**) coronal image showing right parieto-occipital and right cerebellar lesions (white arrows) and left posterior parietal lesion (blue arrow) after three cycles of ipilimumab-nivolumab. (**C**) Post-surgical resection of two symptomatic lesions- right parieto-occipital and right cerebellar lesions with persistent left posterior parietal lesion (arrow). (**D**) Enlarging left posterior parietal lesion post TVEC and pembrolizumab. (**E**) Surgical cavity after resection of left posterior parietal lesion and one month after TMZ treatment. (**F**) Collapsing surgical cavity of left posterior parietal lesion resection with no evidence of recurrent disease in the brain after 19 months of temozolomide initiation.

**Figure 3:**
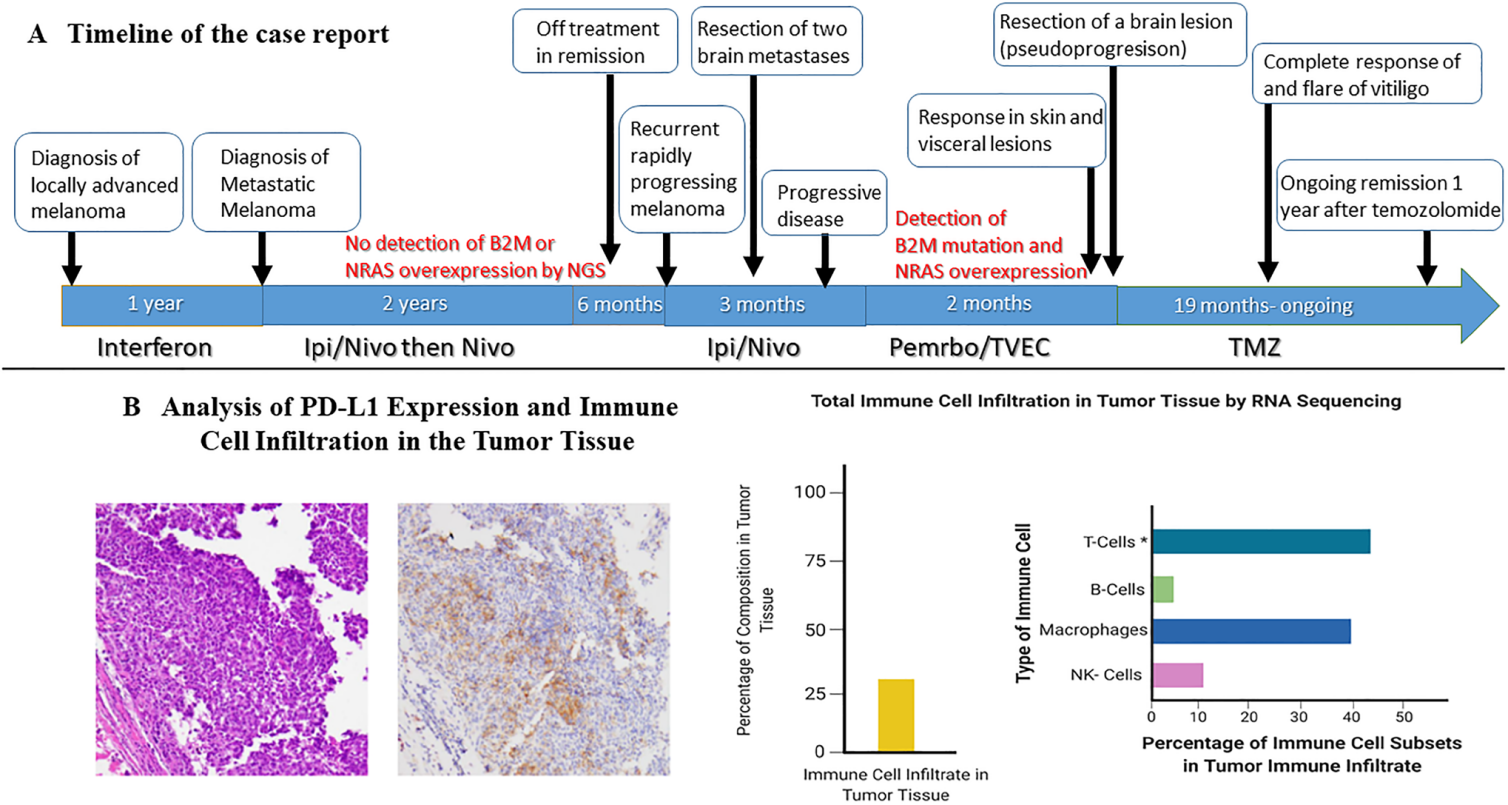
Timeline of the case and changes in lymphocyte count during treatment with temozolomide. (**A**) The timeline of the case report. (**B**) Demonstrates the evidence of an inflammatory tumor status at progression after treatment with ICI, to the left of the panel immunohistochemistry staining demonstrates PD-L1 expression > 30% and to the right RNA sequencing using Tempus xT identified tumor cell infiltration with immune cells composing 27% of the tumor infiltrate with predominance of T-cell infiltration constituting 44% of the infiltrate.

A 33-year-old female presented due to stage IV recurrent metastatic melanoma to the bones, lung, liver and brain. Her medical history was significant for a diagnosis of melanoma in 2015 in the left arm (T3b) with ulceration and Breslow depth of 2.3 mm. She underwent wide local excision and sentinel lymph node biopsy with 2 positive axillary lymph nodes. This was followed by axillary lymph node dissection that showed 0 involved nodes out of 23 total lymph nodes. The patient received high dose adjuvant interferon for 1 year. Surveillance computed tomography (CT) showed multiple liver, breast and lung lesions concerning for metastatic disease 8 months after concluding interferon therapy. Breast biopsy revealed metastatic melanoma and next-generation sequencing (NGS) showed NRAS (p.Q61H) mutation with variant allele fraction (VAF) of 77.4%, IDH1 (p.R132C) mutation with VAF of 52.4%, TERT promoter mutation and no alterations in BRAF, KIT, or B2M. RNA sequencing did not demonstrate overexpression of NRAS. At that time the patient commenced combination immunotherapy with ipilimumab (3 mg/kg) and nivolumab (1 mg/kg) intravenously every three weeks for 4 cycles. CT showed complete response and maintenance nivolumab was continued for 2 years complicated by mild vitiligo on the face during treatment. Six months after stopping maintenance nivolumab, she presented with the chief complaint of numerous rapidly progressing firm subcutaneous nodules in the back, right chest wall and bilateral axilla (Figure 1A). Positron Emission Tomography/Computed Tomography (PET/CT) with [^18^F] fluorodeoxyglucose (FDG) PET showed right apical chest wall osteolytic mass with intrapulmonary invasion, numerous subcutaneous metastatic nodules and nodular metastases involving the left breast, right adrenal gland, retroperitoneal and left perianal areas (Figure 1B). Brain magnetic resonance imaging (MRI) showed enhancing lesions in the left posterior parietal lobe measuring 2.4 × 2.1 × 1.8 cm, right parieto-occipital measuring 3.0 × 2.7 × 2.3 cm (Figure 2) as well as right inferior temporal lesion measuring 8 mm (not shown in the figures). The patient underwent gamma knife radiation to the metastatic brain lesions. Given disease recurrence and prior response to combination immune checkpoint inhibitors, she re-started ipilimumab (3 mg/kg) and nivolumab (1 mg/kg) intravenously every 4 weeks for 4 cycles. The patient completed combination immunotherapy and developed grade 3 autoimmune hepatitis which was treated with glucocorticoids and mycophenolate mofetil. She subsequently developed new neurological symptoms presenting with headache and gait imbalance. Brain MRI showed progression in the right parietal lobe, right posterior cerebellum and the left posterior parietal lesions. She underwent resection of the symptomatic right parietal and right posterior fossa lesions and histopathology confirmed the diagnosis of metastatic melanoma (Figure 1C). This was followed by 30 Gy whole brain radiation over a total of 10 fractions. Repeat NGS analysis of a progressive lung lesion revealed the continued presence of a NRAS (p.Q61H) mutation with VAF of 49.6%, and IDH1 mutation (p.R132C) with VAF of 15.3% along with the interval development of a loss-of-function frameshift mutation in B2M with VAF of 14.5% (Figure 3A case report timeline). The presence of a B2M mutation was concerning for the development of acquired resistance to immunotherapy. We performed RNA sequencing which revealed overexpression of NRAS and tumor immune infiltrate composed of 27% immune cells in the tumor with T-cells constituting 44% (Figure 3B). Immunohistochemistry demonstrated PD-L1 expression of more than 30% in the tumor tissue (Figure 3B).

Talimogene laherparepvec (TVEC) was started with pembrolizumab off-label due to refractory disease with visceral, cutaneous and brain metastases. The patient had good response of the subcutaneous nodules and visceral metastases but had continued radiographic progression of a left parietal lobe lesion which was associated with headache, nausea and vomiting and required left craniotomy with resection (Figure 2). Pathology of the brain showed reactive changes, hyalinized vessels, bland necrosis, and no evidence of malignancy (Figure 1D) most consistent with pseudoprogression. After discussion with the patient, she commenced temozolomide (TMZ) at a dose of 150 mg/m^2^ daily for 5 days out of a 28-day cycle. The patient experienced worsening of pre-existing immune-related vitiligo which manifested by hypopigmented macules and patches on the face, trunk and lower extremities after the 3rd cycle of TMZ (Figure 1E). Repeat PET-CT and brain MRI after 19 months of TMZ initiation showed no evidence of active disease.

## DISCUSSION

Despite the unprecedented advances in the treatment of metastatic melanoma with immune checkpoint inhibitors (ICI), there is a paucity of therapy options in patients with relapsed and refractory disease following progression during treatment with ICI. For example, the rate of progressive disease after initial response to ICI is estimated to be 64% at 5 years during treatment with ipilimumab-nivolumab combination and 79% with pembrolizumab [2, 3]. The development of progressive disease after initial response has been suggested to be mediated by acquired resistance in the tumor microenvironment [4]. This has created an unmet need for better understanding of resistance pathways leading to tumor escape from immune surveillance during treatment with ICI. Our report highlights an example of a patient who had a relapse with high burden and rapidly progressive intracranial and extracranial disease. She responded initially to ipilimumab-nivolumab followed by nivolumab maintenance but later had a recurrence of her disease and was re-challenged with combination ipilimumab-nivolumab. Her visceral and intracranial disease continued to progress despite combination ICI and next-generation sequencing (NGS) revealed the presence of B2M mutation which we believe contributed to the acquired resistance to ICI [5]. Moreover, immunohistochemistry and RNA sequencing of the tumor tissue at progression after treatment with ipilimumab and nivolumab demonstrated an inflamed tumor tissue and abundance of tumor immune cell infiltrate (PD-L1 expression > 30% and immune cell infiltrate of 27% in the tumor, respectively). This however was associated with refractory disease to ICI. Of interest, the overexpression of NRAS in melanoma cells has been demonstrated to be associated with downregulation of MHC class I which consecutively can impair T-cell function in the tumor microenvironment and contribute to resistance to ICI [10–12]. Similarly, B2M mutation has been shown to interrupt MHC class I expression in the tumor microenvironment in melanoma and lead to acquired resistance to ICI [5]. One limitation in our case study is the inability to perform MHC class I expression in the tumor tissue due to unavailable detection methods at our institution. However, the presence of both B2M mutation and NRAS overexpression has been shown as described previously to downregulate MCH class I expression which we believe contributed to the acquired resistance in our patient despite the abundance of immune cell infiltrate including T-cells (44% of the immune infiltrate). The patient had complete and durable response of her visceral metastatic disease after initiating TVEC and pembrolizumab followed by temozolomide (TMZ). The response observed after sequential administration of TVEC and pembrolizumab followed by TMZ raises several important points including the possible role of sequential use of TMZ after immunotherapy resistance due to its immunomodulatory effect and the potential role of overcoming resistance to ICI with TVEC.

### Immunotherapy and Temozolomide in metastatic melanoma

Preclinical and clinical data demonstrated a modulating effect of TMZ on the immune system leading to unleashed anti-tumor immune response. Several mechanisms have been proposed for resistance to ICI such as the interruption of tumor antigen presentation, inhibition of effector T-cell trafficking due to genetic and epigenetic alterations, imbalance between subset of effector and Treg cells and changes in signaling pathways mediated by cytokines [13–16]. Of importance, Treg cells have immunosuppressive effect on anti-tumor immunity and were found to have a role in the resistance to ICI leading to inadequate effector T-cell function against tumor cells [17]. Similarly, the depletion of Tregs have been suggested to restore effector T-cell function and lead to reactivation of anti-tumor immunity [17]. Another important mechanism of resistance in malignant melanoma involves the acquired mutations in the B2M gene leading to decreased expression of MHC class I molecules which are essential in antigen presentation and effective cytotoxic CD8 T-cell function against tumor cells [5].

TMZ is an oral alkylating agent that has shown modest efficacy and tolerability in patients with metastatic melanoma in the era preceding approval of targeted therapies and ICI [8]. In addition, TMZ has a negative effect on lymphocytes leading to lymphodepletion and can affect the number and function of Treg cells [18]. As such, Banissi et al. demonstrated that weight based low dose metronomic administration of temozolomide led to reduction in circulating Treg cells in rat glioma models [9]. Similarly, Ridolfi et al. showed a decrease in circulating Foxp3+ Treg cells after administration of daily temozolomide for 7 days which was further enhanced after the administration of autologous dendritic cell immunotherapy in patients with metastatic melanoma but this observation did not reflect improved clinical outcomes [19]. Whether the reduction of circulatory Treg cells correlates with a decrease in the number of Treg cells recruited in the tumor microenvironment has not been studied. However, the notion that TMZ can lead to a decrease in the number of Treg cells that have been suggested to contribute to the resistance to ICI points to a possible synergistic effect with ICI. The previous concept seems intriguing and has been documented in the literature when TMZ was used with immunotherapy including interleukin-2 (IL-2), dendritic cells (DC) autologous therapy and ICI such as PD-1 and CTLA-4 inhibitors which is summarized in Table 1 [19–23]. The other mechanism by which TMZ is suggested to lead to immunomodulation in the tumor microenvironment is through the modulation of nuclear factor (NF)- kB which increases MHC class I expression in some tumor cells such as glioma [24].

**Table 1:** Studies of Temozolomide with immunotherapy in metastatic melanoma

Tumor Type (front line vs later line therapy)	Immunotherapy	Dose of TMZ	Sequence of Treatment	Outcome	References
Metastatic Melanoma (Front line)	Ipilimumab	200 mg/m^2^	Induction: Ipi IV 10 mg/kg q3w + TMZ 200 mg/m^2^ days 1–4 q3w (4 cycles)	mOS: 24.5 months	Patel SP, et al. 2017 [20]
				mPFS: 5 months	
			Maintenance: Ipi 10 mg/kg q12w + TMZ days 1-5 started on wk 12 and thereafter q4w	ORR 31.2%	
				PFS at 6 months 45%	
Metastatic Melanoma (Later Line)	Pembrolizumab	75 mg/m^2^	TMZ administered daily after progression on pembrolizumab for 6 weeks on, 2 weeks off until response^*^	Patient 1: Complete remission for 2 years	Swami U, et al. 2019 [21]
				Patient 2: Partial remission for 2 months followed by PD	
				Patient 3: durable response for 8 months before PD	
Metastatic Melanoma (Later Line)	HD IL-2	75 mg/m^2^	Patient who did not show response or progressed to HD IL-2 received TMZ for 3 weeks	Rate of Complete or near complete response 67% (6 of 9 patients)	Fateh S, et al. 2010 [22]
Metastatic Melanoma (Front Line)	HD IL-2	75 mg/m^2^	TMZ daily for three weeks followed by HD IL-12	No difference of ORR or durable response compared to single use of HD IL-2	Tarhini AA, et al. 2008 [23]
Metastatic Melanoma (Later Line)	Autologous dendritic cells and IL-2	75 mg/m^2^	TMZ for 7-14 days prior to DC therapy and IL-2^**^	No improvement in clinical outcome	Ridolfi, et al. 2013 [19]
				Decreased Treg cells	

^*^Other treatments were administered to some patients including ipilimumab and CMP-001. ^**^some patients in this study received CTLA-4 inhibitors. PD: progressive disease, TMZ: temozolomide, Ipi: ipilimumab, HD IL-2: High dose interleukin-2, mOS: median overall survival, mPFS: median progression free survival, ORR: overall response rate, DC: dendritic cell, Treg cells: T- regulatory cells.

Notably, sequential use of TMZ with ipilimumab in melanoma patients showed improved progression free survival rates at 6 months compared to ipilimumab alone which further support the previous notion [20]. Our patient had exacerbation of vitiligo which was a skin immune related adverse event (IRAEs), mainly at the sites of prior TVEC injections, three months after initiation of TMZ and despite being off ICI. This phenomenon is consistent with the observation that oncolytic adenovirus with TMZ can induce autophagy and antitumor immune responses [25]. In a similar report, Swami et al. demonstrated complete and durable remission with the use of metronomic TMZ in a patient with metastatic melanoma after refractory disease to pembrolizumab and the observed response was associated with the development of vitiligo [21]. In fact, development of vitiligo has been shown to correlate with improved response and prognosis in patients with metastatic melanoma treated with ICI [26]. As such, we believe that the exacerbation of skin IRAE in our patient during treatment with TMZ was associated with the maintained response. This raises a question on whether TMZ interaction with lymphocyte subsets could have led to induction of autoimmunity and restoration of anti-tumor immune response. However, whether TMZ could overcome B2M mutation resistance requires further validation. Limitations to the hypothesis of a possible benefit of TMZ with immunotherapy in the reviewed studies include the small number of subjects included in these studies, different dosing and sequence of TMZ with immunotherapy that were used in investigations reported in the literature (summarized in Table 1) and lack of mechanistic insight on the role of Treg cells in the tumor microenvironment and mechanisms of resistance to ICI in metastatic melanoma.

### TVEC and immunotherapy in metastatic melanoma

TVEC exerts its effect on injectable cutaneous melanoma sites and has been approved for unresectable and metastatic melanoma with small number of visceral metastases [27]. Also, TVEC has been suggested to overcome resistance to ICI [28]. Despite many mechanisms of resistance that contribute to failure to ICI in metastatic melanoma, it is hypothesized that type I interferon pathway remains intact for many of these patients such as our patient who had no evidence of loss of downstream signaling through JAK1/2 gene modifications [28]. Hence, oncolytic viral injection of cancer cells can lead to upregulation of cytokines such as type I IFN which can activate natural killer cells. This can lead to the killing of target cells despite downregulated MHC class I expression due to mutations such as the loss of B2M, leading to restoration of anti-tumor immunity which could explain the systemic response observed in some the patients who receive TVEC. The activation of type I IFN pathway by oncolytic viral injection of malignant cells can explain the circumventing mechanism by which TVEC can bypass resistance to ICI and lead to restoration of anti-tumor immunity [29]. The response observed in our patient with regression of her cutaneous and visceral disease despite having acquired resistance to ICI could have been secondary to the administration of TVEC and pembrolizumab. Our patient’s intracranial and extracranial response to TVEC and pembrolizumab is similar to the report by Blake et al. [30] but our patient had more durable response which supports a possible role of adding TMZ in preventing further development of the metastatic disease.

Of interest, the patient had detectable isocitrate dehydrogenase (IDH) mutation which has been reported to be present with a frequency of 6% in malignant melanoma [31]. Interestingly, IDH mutated cancer cells are sensitive to TMZ but this has not been illustrated well in the case of melanoma [32]. The lack of evidence on the role of IDH mutations in tumurogenesis in metastatic melanoma and the effect of TMZ on IDH mutated melanoma cells poses intriguing questions on any possible interactions and benefit of using TMZ in patients with metastatic melanoma harboring IDH mutations.

Finally, it is important to highlight the limitation in our case study because of the inability to perform MHC class I expression at progression, which we believe was downregulated due to the presence of B2M mutation and NRAS overexpression. Moreover, it is difficult to conclude that the addition of TMZ had an added benefit to TVEC and pembrolizumab. However, TMZ has efficacy in intracranial tumors because of its ability to cross the blood-brain barrier which could have contributed to the response observed. In addition, development of vitiligo is known to be associated with a restored immune response in ICI treated melanoma patients, and this was observed in our patient after starting TMZ which we believe contributed to restoration of anti-tumor immune response.

## CONCLUSIONS

We report the first case to our knowledge of a successful administration of TVEC with pembrolizumab followed by TMZ in a patient with ICI refractory (intracranial and extracranial) metastatic melanoma with acquired B2M mutation. This report highlights the need for further investigation on whether TVEC and TMZ have a role in immunotherapy refractory metastatic melanoma. This report should be interpreted with caution as more preclinical and clinical studies are essential to confirm whether the presence of acquired B2M mutation can be broken with the use of TVEC and pembrolizumab followed by TMZ.
