# Addendum: Successful administration of sequential TVEC and pembrolizumab followed by Temozolomide in immunotherapy refractory intracranial metastatic melanoma with acquired B2M mutation

**DOI:** 10.18632/oncotarget.28365

**Published:** 2023-02-11

**Authors:** Karam Khaddour, Joshua Dowling, Jiayi Huang, Martha Council, David Chen, Lynn Cornelius, Tanner Johanns, Sonika Dahiya, George Ansstas

**Affiliations:** ^1^Department of Medicine, Division of Medical Oncology, Washington University School of Medicine, St. Louis, Missouri, USA; ^2^Department of Neurological Surgery, Washington University School of Medicine, St. Louis, Missouri, USA; ^3^Department of Radiation Oncology, Washington University School of Medicine, St. Louis, Missouri, USA; ^4^Division of Dermatology, Washington University School of Medicine, St. Louis, Missouri, USA; ^5^Division of Neuropathology, Department of Pathology and Immunology, Washington University School of Medicine, St. Louis, Missouri, USA

**This article has an addendum:** Informed patient consent was obtained.

Original article: Oncotarget. 2020; 11:4836–4844. 4836-4844. https://doi.org/10.18632/oncotarget.27848

